# The effect of gender role stress on psychological distress in women with breast cancer after mastectomy

**DOI:** 10.1007/s00520-026-10722-x

**Published:** 2026-06-06

**Authors:** Sakine Yılmaz, Serap Açıkgöz, Osman Çağdaş Erdem

**Affiliations:** 1 Department of Midwifery, Faculty of Health Sciences, Karatekin University, Çankırı, Türkiye; 2 Department of Public Health Nursing, Faculty of Health Sciences, Karatekin University, Çankırı, Türkiye; 3 Emergency Department, Dr. Abdurrahman Yurtaslan Ankara Oncology Training and Research Hospital, University of Health Sciences, Ankara, Türkiye

**Keywords:** Breast cancer, Gender role, Mastectomy, Psychological distress, Stress

## Abstract

**Purpose:**

Breast cancer is also the most common cancer in women in Turkey. The breast is closely linked to female identity and the feminine gender role. It plays an important role in a woman's body image, self-perception, and consequently**,** her psychological well-being. One of the variables that is not sufficiently recognized in breast cancer treatments, especially after mastectomy, is gender roles and the stress stemming from these roles. This study examined the impact of gender role stress on psychological distress in women.

**Methods:**

A descriptive cross-sectional study was conducted with 264 women with breast cancer who attended the surgical oncology outpatient clinic of a state hospital in Türkiye. Data were collected using the Personal Information Form, the Female Gender Role Stress Scale, and Kessler's Psychological Distress Scale. One-way Analysis of Variance, Kruskal–Wallis H Test, Independent sample t-test, and Pearson correlation coefficient were used in data analysis.

**Results:**

Women with breast cancer were found to experience high levels of gender role stress and psychological distress. Women who held bachelor's degrees, had a nuclear family structure, and whose spouses held bachelor's degrees experienced high levels of gender role stress and psychological distress. Gender role stress experienced by women with breast cancer was not significantly associated with psychological distress.

**Conclusions:**

The results reveal the importance of evaluating gender role stress alongside psychological health in women with breast cancer after mastectomy. Nurses should consider psychological challenges and coping strategies alongside gender roles when evaluating women with post-mastectomy breast cancer holistically in terms of health.

## Introduction

Breast cancer is an increasing global health, gender, socioeconomic, and equity challenge [1]. Breast cancer (BC) is the most common type of cancer in women worldwide (11.7%) and in Türkiye (23.9%) [2]. Treatment of BC can include a combination of surgery, chemotherapy, radiation, and hormone therapy. Mastectomy, which is the removal of the entire breast affected by cancer, is the most commonly used surgical treatment method. Mastectomy treatment can improve survival rates, but the process can also lead to physical and emotional problems for women [3]. After mastectomy surgery, women have difficulty accepting the physical changes to their bodies following surgery (scarring and breast loss) and face many challenges, such as a disrupted body image and loss of self-confidence. This process, which is difficult to accept and a source of serious stress, causes problems affecting the mental health of women and their family members. These emotional disruptions following the diagnosis affect the woman's self-care, her treatment compliance, the severity and course of cancer over time, her response to treatment, and her quality of life [4–6]. Health-related quality of life is affected by multiple complex structural components, including sociodemographic characteristics, disease characteristics (cancer location, prognosis, pain), biological determinants, spirituality, social support, psychological status, and lifestyle-related factors [7]. All these affect the quality of life and also affect coping with cancer and its consequences [6].

The concept of "gender" refers to the status, roles, and position of women or men in the society in which they live, the societal perspective on the individual, the duties and responsibilities of the individual, and the societal expectations of the individual [8]. Gender role stress, on the other hand, is defined as the stress that arises when a person fails to meet what is expected of them due to their assigned gender roles [9]. Understanding the role of gender in physical functioning in women with breast cancer is crucial for several reasons (participation in physical activity, compliance with rehabilitation programs, caregiving responsibilities, etc.). Gender roles and norms in breast cancer shape individuals' participation in physical activity and their compliance with post-treatment rehabilitation programs. Additionally, gender roles can intensify the caregiving burden, with women frequently taking on more caregiving responsibilities, which can exacerbate emotional stress [10]. One of the important and under-recognized variables in coping with breast cancer, especially a post-mastectomy breast cancer diagnosis, is gender roles and the stress that results from these roles [3]. A review exploring gender-related experiences of cancer survivors through creative arts found that gendered psychosocial challenges (e.g., changes in roles and responsibilities due to cancer) and changes to the physical body after treatment (e.g., hair loss or breast loss) impacted survivors' experience of their gendered self-image [11]. The responsibilities women feel obliged to fulfill due to gender roles, the societal impositions placed on women**,** and expectations of women are stressors for women [8, 12].

Psychological distress is likely to occur when an individual encounters stressors that they are unable to cope with. Psychological distress is a state of suffering experienced by an individual in the recent weeks, characterized by symptoms of depression and anxiety, including impairments in normal or healthy functioning. Psychological distress can be manifested through many symptoms, such as feeling distressed, hopeless, worthless, or anxious most of the time, disruption of eating and sleeping patterns, chronic fatigue, avoidance of social activities, memory problems, and decreased sexual pleasure [13, 14]. Emotional functioning can be influenced by gender dimensions in several ways. Social expectations and gender norms can shape how individuals cope with cancer and seek emotional support. Women generally make greater use of emotional and social support networks, but they may struggle with societal pressures to maintain feminine roles, which can hinder their emotional recovery. Additionally, gender roles can increase the burden of caregiving, as women typically assume more caregiving responsibilities, which can intensify emotional stress [10, 15]. Parenting roles, decision-making power, and other marital or partner roles such as emotional, social, economic, and administrative household tasks can significantly affect the quality of life and coping mechanisms of women with cancer who face health problems [16].

According to feminist theory, the breast is recognized as a representation of a woman's femininity. The breast is, therefore, closely linked to female identity and feminine gender roles. Objectification theory links the extent to which a woman's body matches or does not match societal standards with her self-worth. Studies report that feminine gender role orientation plays an important role in women's body image and sense of self after treatment and, thus, in their psychological well-being [17–19]. Gender influences social roles, support networks, and societal expectations, which can shape the social experiences of cancer patients in significant ways. Traditional gender roles can also place an additional burden on women, who may continue to shoulder caregiving and domestic responsibilities even while coping with their own illness [3]. BC is also the most common cancer in women in Türkiye [4]. However, in Türkiye, there is no study investigating the relationship between gender role-related stress and psychological distress in post-mastectomy women. This study was conducted to examine the effect of gender role-related stress levels on psychological distress in women with breast cancer after mastectomy. It is thought that the results of the study will increase the awareness of health professionals about the stress caused by gender roles in women with breast cancer and guide the counseling services to be provided in this regard.

### Research questions

- What is the level of gender role stress and psychological distress in women after mastectomy?
- According to which characteristics do gender role stress and psychological distress levels of post-mastectomy women differ?
- What is the relationship between gender role stress and psychological distress?

## Methods

### Study design and participants

**Fig. 1:**
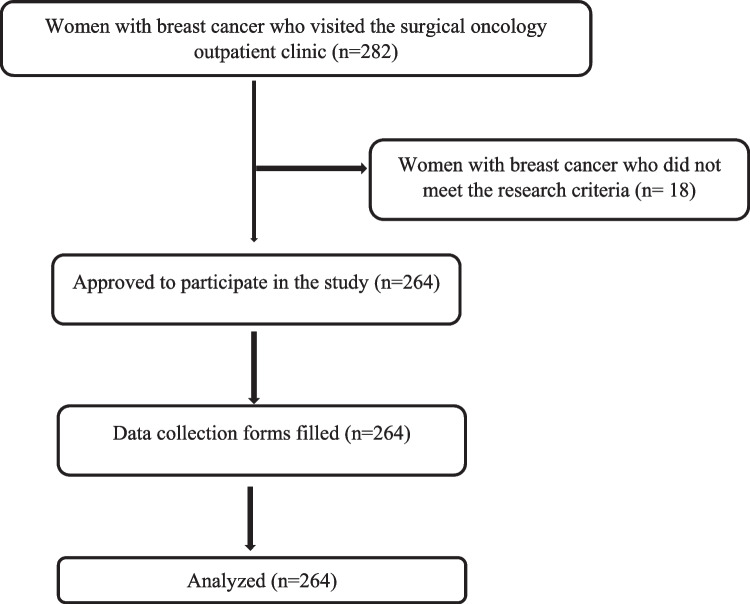
Flow chart of the study

The study employed a descriptive, cross-sectional design. The study was conducted in the surgical oncology outpatient clinic of a state hospital in Türkiye. In the sample selection, the random sampling method was used from among non-probability sampling methods. The sample comprised women over the age of 18 who were diagnosed with breast cancer and underwent mastectomy, had no communication difficulties, had no diagnosis of neuropsychiatric disorder, and agreed to participate in the study. The exclusion criteria were requesting to leave the study and not completing the data collection tools completely. Studies with a similar theoretical framework that relate psychosocial outcomes to genderization processes were reviewed in the literature. In the sample calculation, we used Trachtenberg et al.'s study (2022) as a reference. In this context, the magnitude of the relationships reported in the referenced study [3] was moderate (based on the correlation coefficient between the Gender Role Socialization Scale and the Functional Assessment of Cancer Therapy-Breast, approximately r ≈ 0.40–0.45), and the effect size we expected clinically and methodologically in our analyses was also at this level. Therefore, in our post hoc power analysis, r = 0.45 was used as the target effect size, α = 0.05 as the significance level, and H0: r = 0 as the assumption. Using the data from a previous study [3] this study was completed with 264 women with 95% confidence (1-α), and the power of the test (1-β) was determined as 100% as a result of post hoc power analysis. Eighteen women with breast cancer declined to participate in the study, which was completed with 264 women (Fig. 1).

### Data collection tools

The data were collected by the researchers using the "Personal Information Form," "Women's Gender Role Stress Scale," and "Kessler's Psychological Distress Scale".

The personal information form was prepared by researchers based on the literature and in line with three expert opinions. This form consists of two sections and 16 questions. In the first part of the form, there are 10 questions related to sociodemographic characteristics such as age, marital status, and educational status. In the second part, there are six questions about women's characteristics related to breast cancer, such as time of diagnosis and disease stage [3, 4, 8].

The Women's Gender Role Stress Scale was developed by Koç, Haskan-Avcı, and Bayar (2017). There are a total of 20 items on the scale. The score that can be obtained from the five-point Likert-type scale (1 = Not stressful for me at all, 2 = Slightly stressful for me, 3 = Undecided, 4 = Stressful for me, 5 = Very stressful for me) ranges from 20 to 100. A high score indicates that the individual experiences gender role stress, while a low score indicates that the individual does not experience gender role stress. The Cronbach alpha coefficient of the scale was determined as 0.92 [8]. In this study, the Cronbach's alpha coefficient of the scale was determined as 0.95.

Kessler's Psychological Distress Scale (K10-PDS) was developed by Kessler et al. (2003), and the Turkish validity and reliability study was conducted by Altun, Özen, and Kuloğlu (2019). The five-point Likert-type scale consists of 10 questions and measures the level of depressive symptoms experienced by a person within four weeks. The scale has a minimum score of 10 and a maximum score of 50. Higher scores indicate higher psychological distress. K10 total score interpretation is as follows: A score between 10 and 19 points indicates probably good mental health; 20 to 24 points indicates possible mild mental illness; 25 to 29 points indicates possible moderate mental illness; and 30 to 50 points indicates possible severe mental illness. The Cronbach's alpha coefficient of the scale is 0.95 [20]. In this study, the Cronbach's alpha coefficient of the scale was 0.89.

### Data collection

The data were collected between December 2022 and July 2023 through face-to-face interviews with women who attended the surgical oncology outpatient clinic and met the inclusion criteria. Each interview lasted an average of 15–20 min.

### Ethical considerations

Written approval was obtained from the Çankırı Karatekin University Ethics Committee (October 25, 2022, no. 3) and from the participating institution [November 29, 2022, no**.** 165]. Before inclusion in the study, the women were informed about the purpose and method of the study. Informed consent was obtained from women who agreed to participate in the study. This research was conducted in accordance with the Helsinki Declaration.

### Data analysis

The SPSS statistical package (version 23.0; SPSS, Inc., USA) was used to analyze the data. Number, percentage, mean (minimum, maximum), and standard deviation values were used for descriptive statistics. Normality of distribution was analyzed using skewness and kurtosis coefficients (± 3). The Independent Sample t-test was used for paired groups, and One-Way Analysis of Variance was used for groups of three or more. Multiple comparisons were made with the Duncan and Tamhane tests. The Kruskal Wallis H Test was used to compare scale scores that did not fit the normal distribution in groups of three or more, and Dunn's test was used for multiple comparisons. The Pearson correlation coefficient was used to examine the relationship between variables and scale scores that fit the normal distribution. The statistical significance level was taken as *p* < 0.05.

## Results

### Participant characteristics

The mean age of the women who participated in the study was 49.85 ± 10.03 years. Among the women, 79.5% were married, 29.9% were high school graduates, and 56.8% were housewives. The proportion of those who stated that their income was less than their expenses was 48.9%, those who had a nuclear family was 74.6%, and those who stated that they lived in the province was 40.5%. Among the women, 92.8% stated that there was no external interference in family relations, and 76.9% stated that they did not have chronic diseases. Of the women's spouses, 28.4% were primary school graduates (Table 1).

**Table 1:** Women's descriptive characteristics (*n* = 264)

Characteristics	Mean ± Sd	Medıan (Mın-Max)
Age	49.85 ± 10.03	50.5 (26—77)
	*n*	*%*
Marital Status		
Married	210	79.5
Single	54	20.5
Education status		
Illiterate	9	3.4
Literate	39	14.8
Primary education	65	24.6
High School	79	29.9
University	72	27.3
Profession		
Housewife	150	56.8
Working	91	34.5
Retired	23	8.7
Income level		
Income less than expenditure (Low)	129	48.9
Income equal to expenditure (Medium)	126	47.7
Income more than expenditure (High)	9	3.4
Family type		
Nuclear family (mother, father, and children)	197	74.6
Extended family (mother, father, children and relatives)	67	25.4
Place of residence		
Village	37	14.0
Town	38	14.4
District	82	31.1
Province	107	40.5
Family intervention		
Yes	19	7.2
No	245	92.8
Chronic disease status		
Yes	61	23.1
No	203	76.9
Spouse education status		
Those without a spouse	53	20.1
Illiterate	2	0.8
Literate	16	6.1
Primary education	75	28.4
High School	61	23.1
University	57	21.6

Mean ± SD: Mean ± Standard deviation. Min—Max: Minimum–maximum

### Participant characteristics related to breast cancer

The time of diagnosis was less than one year for 52.3% of the women; 50.4% had first-stage breast cancer, and 71.6% had one breast removed. Within 0–2 months after diagnosis, 37.1% of the women underwent mastectomy surgery, 55.7% received chemotherapy, and 30.3% had follow-up visits every three months (Table 2).

**Table 2:** Women's characteristics related to breast cancer (*n* = 264)

Variables	*N*	*%*
Diagnosis Time		
Less than a year	138	52.3
1–5 years ago	101	38.3
5–10 years ago	21	8
More than 10 years	4	1.5
Phase		
Phase 1	133	50.4
Phase 2	86	32.6
Phase 3	45	17
Surgery Type		
Both breasts were removed (Total Mastectomy)	75	28.4
One breast was removed (Partial)	189	71.6
Operation Time		
0–2 months	98	37.1
3–5 months	68	25.8
6–9 months	46	17.4
10–12 months	16	6.1
More than a year	36	13.6
Treatment Applied		
Chemotherapy	147	55.7
Radiotherapy	15	5.7
Chemoradiotherapy	77	29.2
Not receiving treatment	25	9.5
Control Frequency		
Once a month	47	17.8
Every three months	80	30.3
Every six months	66	25
Once a year	53	20.1
Other*	18	6.8

^*^There is no routine

### Women's gender role stress scale and K10-PDS scores

The participants' mean score on the Female Gender Role Stress Scale was 77.95 ± 17.84, and the mean score on the K10-PDS was 35.17 ± 7.68 (Table 3).

**Table 3:** Women's gender role stress scale and K10-PDS score distributions (*n* = 264)

Scales	Mean ± SD	Median (Min–Max)
Women's gender role stress scale	77.95 ± 17.84	78 (20—100)
Psychological distress scale	35.17 ± 7.68	36 (15—50)

Mean ± SD: Mean ± Standard deviation. Min—Max: Minimum–maximum

### Scale scores based on women's characteristics

In Table 4, the mean gender role stress score of women with bachelor's degrees (91.49 ± 12.12) was statistically significantly higher than the others (*p* < 0.001). The mean gender role stress score of working women (90.97 ± 11.04) was statistically significantly higher than the others (*p* < 0.001). The mean gender role stress score of women whose income was higher than their expenses (94.89 ± 8.49) was statistically significantly higher than the others (*p* < 0.001). The mean gender role stress score of women with nuclear family type (80.9 ± 17.19) was statistically significantly higher than those with extended family (69.3 ± 16.98) (*p* < 0.001). The mean gender role stress score of women living in the province (89.37 ± 13.46) was statistically significantly higher than the others (*p* < 0.001). The mean gender role stress score of women whose spouses had undergraduate degrees was found to be statistically significantly higher than those whose spouses were literate or had primary school education (*p* < 0.001).

**Table 4:** Comparison of scale scores based on women's characteristics (*n* = 264)

Variables	Women's Gender Role Stress Scale			K10-PDS	
	Mean ± SD	Median (Min–Max)		Mean ± SD	Median (Min–Max)
Marital Status					
Married	77.48 ± 17.42	78 (35—100)		35.29 ± 7.77	37 (15—48)
Single	79.8 ± 19.42	81.5 (20—100)		34.69 ± 7.4	34 (18—50)
Test Statistic	*t* = −0.850; p = 0.396			*t* = 0.516; 0.607	
Education Status					
Illiterate	58.78 ± 14.62^a^	60 (35—78)		30.67 ± 6.22^a^	30 (22—42)
Literate	63.18 ± 13.45^ab^	60 (20—94)		31.54 ± 7.65^b^	33 (15—50)
Primary education	69.58 ± 14.78^b^	66 (46—100)		34.46 ± 6.48^ab^	35 (18—47)
High School	81.99 ± 15.95^c^	83 (38—100)		35.75 ± 7.9^b^	37 (18—47)
University	91.49 ± 12.12^d^	98 (54—100)		37.69 ± 7.71^b^	39 (17—49)
Test Statistic	*F* = 37.798; *p* < 0.001			*F* = 5.496; *p* < 0.001	
Profession					
Housewife	70.3 ± 17^a^	66 (20—100)		34.37 ± 7.42^b^	35 (15—50)
Working	90.97 ± 11.04^b^	96 (56—100)		35.77 ± 8.42^ab^	38 (17—49)
Retired	76.39 ± 15.67^a^	76 (45—100)		38 ± 5.29^a^	38 (27—47)
Test Statistic	*F* = 66.27; *p* < 0.001			*F* = 4.271;*p* = 0.018	
Income Level					
Income less than expenditure	72.77 ± 18.38^c^	69 (20—100)		35.92 ± 8.1	35 (15—50)
Income matches expenditure	82.06 ± 15.92^b^	83 (43—100)		34.52 ± 7.19	36.5 (17—48)
Income more than expenditure	94.89 ± 8.49^a^	98 (80—100)		33.33 ± 8.03	35 (18—43)
Test Statistic	*F* = 24.249;* p* < 0.001			*F* = 1.325; *p* = 0.268	
Family Type					
Nuclear Family	80.9 ± 17.19	82 (38—100)		36.15 ± 7.74	38 (17—50)
Extended Family	69.3 ± 16.98	63 (20—100)		32.28 ± 6.8	33 (15—48)
Test Statistic	*t* = 4.786; *p* < 0.001			*t* = 3.637; *p* < 0.001	
Place of residence					
Village	59.51 ± 13.21^c^	58 (20—89)		33.14 ± 7.48^a^	33 (18—50)
Town	72.11 ± 16.55^b^	68 (48—100)		32.97 ± 6.39^a^	34 (20—45)
District	74.09 ± 15.32^b^	76 (38—100)		34.7 ± 7.51^ab^	36 (15—46)
Province	89.37 ± 13.46^a^	97 (54—100)		37.01 ± 7.96^b^	38 (17—49)
Test Statistic	*F* = 52.225; *p* < 0.001			*F* = 4.196; *p* = 0.006	
Spouse Education Status					
Those without a spouse	79.74 ± 19.6^ab^	81 (20—100)		34.94 ± 7.22^bc^	34 (18—50)
Literate	60 ± 14.07^c^	58 (35—79)		31.38 ± 7.45^a^	34 (15—40)
Primary education	71.87 ± 14.89^bc^	72 (48—100)		33.48 ± 7.41^ab^	35 (18—47)
High School	80.05 ± 18.44^ab^	83 (38—100)		37.05 ± 6.59^c^	38 (18—46)
University	87.11 ± 13.88^a^	90 (51—100)		37.11 ± 8.55^c^	38 (17—48)
Test Statistic	*F* = 15.763; *p* < 0.001			*F* = 3.925; *p* = 0.004	
Chronic Disease					
Yes	76.66 ± 16.24	76 (45—100)		32.72 ± 7.75	33 (18—46)
No	78.34 ± 18.31	80 (20—100)		35.9 ± 7.53	37 (15—50)
Test Statistic	*t* = −0.648; *p* = 0.518			*t* = −2.874; *p* = 0.004	
Family Intervention					
Yes	78.89 ± 19.04		79 (35—100)	27.84 ± 11.52	20 (15—48)
No	77.88 ± 17.78		78 (20—100)	35.73 ± 7.02	37 (17—50)
Test Statistic	*t* = 0.238; *p* = 0.812			*t* = −2.944;* p* = 0.008	

Mean ± SD: Mean ± Standard deviation. Min—Max: Minimum–maximum. t: Independent Samples t Test; F: One-Way Analysis of Variance; a-d There is no difference between groups with the same letter

The mean psychological distress score of literate women, as well as high school, and undergraduate graduates was statistically significantly higher than those who were illiterate (*p* < 0.001). The mean psychological distress score of retired women (38 ± 5.29) was statistically significantly higher than that of housewives (34.37 ± 7.42) (p = 0.018). The mean psychological distress score of women with nuclear family type (36.15 ± 7.74) was statistically significantly higher than those with extended family (32.28 ± 6.8) (p < 0.001). The mean psychological distress score of women living in the city (37.01 ± 7.96) was statistically significantly higher than those living in villages (33.14 ± 7.48) and towns (32.97 ± 6.39) (p = 0.006). The mean psychological distress score of those whose spouses had undergraduate degrees (37.11 ± 8.55) was statistically significantly higher than those whose spouses had primary education or were literate (p = 0.004). The mean psychological distress score of those without chronic disease (35.9 ± 7.53) was statistically significantly higher than those with chronic disease (32.72 ± 7.75) (p = 0.004). The mean psychological distress score of women who did not experience domestic intervention (35.73 ± 7.02) was statistically significantly higher than those who experienced domestic intervention (27.84 ± 11.52) (p = 0.**0**08; Table 4 ).

### Scale scores based on women's characteristics related to breast cancer

In Table 5, the mean gender role stress score of those whose post-diagnosis surgery time was 3–5 months (70.12 ± 15.67) was similar to that of those whose post-diagnosis surgery time was 6–9 months (76.48 ± 17.07) (*p* > 0.05), while a statistically significant difference was found in the mean scores of the other groups (*p* < 0.001). The mean gender role stress scores of women who had monthly (82.38 ± 17.09) and quarterly (81.14 ± 16.8) check-ups were statistically significantly higher than those who had annual check-ups (70.72 ± 15.96) (*p* = 0.004).

The mean psychological distress score of women with stage 1 breast cancer (37.16 ± 7.52) was significantly higher than those with stage 2 (32.63 ± 7.71) and stage 3 (34.13 ± 6.55) (*p* < 0.001). The mean psychological distress score of women with partial mastectomy (36.44 ± 7.12) was significantly higher than those with total mastectomy (31.96 ± 8.16) (*p* < 0.001). The mean psychological distress score of women who did not receive any treatment other than surgery (41.24 ± 4.06) was significantly higher than those who received chemotherapy (35.8 ± 7.02) or chemoradiotherapy (32.13 ± 8.28) (p < 0.001). The mean psychological distress scores of those who went for follow-up visits every three months (36.74 ± 7.19), every six months (36.73 ± 8.29), and at other times (38.83 ± 6.87) were statistically significantly higher than those who went for follow-up visits every year (30.55 ± 6.3) (p = 0.004; Table 5).

**Table 5:** Comparison of scale scores based on women's characteristics related to breast cancer (*n* = 264)

Variables	Women's Gender Role Stress Scale			K10-PDS			
	Mean ± SD	Median (Min–Max)		Mean ± SD			Median (Min–Max)
Diagnosis Time							
Less than a year	78.49 ± 18.4	78 (35—100)		36,07 ± 6,82			37 (17—48)
1–5 years ago	78.94 ± 16.26	80 (43—100)		33,88 ± 8,22			35 (18—49)
5–10 years ago	71.1 ± 20.37	74 (20—100)		34,67 ± 9,93			37 (15—50)
More than 10 years	70.5 ± 20.09	63 (56—100)		39 ± 5,48			39 (33—45)
Test Statistic	${\chi }^{2}$= 3.454; *p* = 0.327			${\chi }^{2}$= 3.826; *p* = 0.281			
Phase							
Phase 1	79.32 ± 18.68	80 (20—100)		37.16 ± 7.52^b^			38 (17—50)
Phase 2	77 ± 17.04	78 (35—100)		32.63 ± 7.71^a^			33 (15—49)
Phase 3	75.73 ± 16.77	75 (52—100)		34.13 ± 6.55^a^			35 (20—45)
Test Statistic	*F* = 0.863; *p* = 0.423			*F* = 10.241; *p* < 0.001			
Surgery Type							
Both breasts were removed (Total Mastectomy)	79.19 ± 18.07	80 (20—100)		31.96 ± 8.16			33 (15—50)
One breast removed (Partial)	77.47 ± 17.77	78 (35—100)		36.44 ± 7.12			37 (18—49)
Test Statistic	*t* = 0.706;* p* = 0.481			*t* = −4.42; *p* < 0.001			
Operation Time							
0–2 months	81.52 ± 18.93^b^	85 (20—100)		35.62 ± 9.01			37 (17—49)
3–5 months	70.12 ± 15.67^a^	66 (35—100)		35.72 ± 7.09			36.5 (15—50)
6–9 months	76.48 ± 17.07^ab^	76 (48—100)		36.28 ± 4.51			35 (26—47)
10–12 months	82.94 ± 16.93^b^	84 (45—100)		33.13 ± 6.72			34 (18—42)
More than a year	82.72 ± 15.43^b^	81.5 (51—100)		32.36 ± 7.99			35.5 (18—45)
Test Statistic	*F* = 5.670; *p* < 0.001			*F* = 2.192; *p* = 0.078			
Treatment Applied							
Chemotherapy	75.8 ± 17.25		76 (35—100)	35.8 ± 7.02^c^	36 (15—49)		
Radiotherapy	81.2 ± 18.6		94 (51—100)	34.4 ± 8.53^abc^	37 (18—47)		
Chemoradiotherapy	79.83 ± 17.28		80 (20—100)	32.13 ± 8.28^b^	33 (17—50)		
No	82.92 ± 21.38		98 (38—100)	41.24 ± 4.06^a^	42 (33—48)		
Test Statistic	*F* = 1.83; *p* = 0.142			*F* = 18.771; *p* < 0.001			
Control Frequency							
Once a month	82.38 ± 17.09^b^		86 (46—100)	34.11 ± 7.1		35 (18—48)^ab^	
Every three months	81.14 ± 16.8^b^		83 (45—100)	36.74 ± 7.19		39 (15—46)^a^	
Every six months	77.73 ± 18.42^ab^		80 (20—100)	36.73 ± 8.29		38 (18—50)^a^	
Once a year	70.72 ± 15.96^a^		68 (35—100)	30.55 ± 6.3		31 (17—48)^b^	
Other	74.39 ± 21.57^ab^		72.5 (38—100)	38.83 ± 6.87		38.5 (17—47)^a^	
Test Statistic	*F* = 3.888;* p* = 0.004			${\chi }^{2}$= 40.375; *p* < 0.001			

Mean ± SD: Mean ± Standard deviation. Min—Max: Minimum–maximum. *t:* Independent Samples t Test; *F:* One-Way Analysis of Variance; a-d There is no difference between groups with the same letter

### Relationship between age and scale scores

A statistically significant weak negative correlation was found between women's age and Gender Role Stress Scale scores (r = −0.375; *p* < 0.001). A statistically significant very weak negative correlation was found between women's age and K10-PDS scores (r = −0.138; p = 0.025). There was no statistically significant relationship between the Gender Role Stress Scale scores and K10-PDS scores (p = 0.991; Table 6).

**Table 6:** Analysis of the relationship between age and scale scores

Scales	Age	Women Gender Role Stress Scale
Women Gender Role Stress Scale	*r* = −0.375; *p* < 0.001	
K10-PDS	*r* = −0.138; *p* = 0.025	*r* = 0.001; *p* = 0.991

*r:* Pearson Correlation Coefficient

## Discussion

Breast cancer cases increased from 2 million to 2.2 million between 2013 and 2020 [2]. These results emphasize the global burden of breast cancer. Therefore, there is a need to understand women's long-term experiences of treatment and post-treatment and to continue efforts to address the problems that the process brings with it [19]. Women with breast cancer whose bodies are affected, including their breasts, hair, vaginal tissues, fertility status, energy levels, and weight, may have difficulty meeting idealized heteronormative standards of femininity and appearance [3]. Women who uphold traditional gender role expectations and attitudes tend to internalize beauty standards and invest more in their physical appearance. Research has found that women with breast cancer who invest more in physical appearance have more difficulty adapting post-treatment compared to those who invest less. At the same time, the self-esteem, body image, and quality of life of these women were found to be poor [21–23]. In this regard, it is important to evaluate gender role stress and psychological distress in women with breast cancer. The results of this study were discussed with the results of similar studies since no study on gender role stress and psychological distress in women with breast cancer was found.

### Gender role stress in women with breast cancer

Body image is fundamental to young women's self-esteem. Although conservative surgery often provides acceptable cosmetic results in women, mastectomy is associated with disfigurement and reduced femininity and motherhood. This may also lead to anxiety about not being accepted by the woman's spouse and the threat of a break-up of the marriage [24, 25]. In this study, it was determined that women with breast cancer experienced high levels of gender role stress. Everaars et al. (2021) found that reminders such as loss of breast and sexual function increased sadness and negative emotions in women with breast cancer even years after treatment [26]. In a study conducted with women with gynecologic cancer, it was determined that gender role attitude was moderate and did not affect quality of life [27]. In studies conducted on healthy women, it was similarly reported that gender socialization had devastating effects on body and self-image, depression, stress, embodiment, and eating disorders [3, 28]. These differences between the findings of the studies may be due to the individual characteristics of the sample groups and the different cultural characteristics of the societies in which they live. In this study, more than half of the women in the study group were newly diagnosed with breast cancer (52.3%), received chemotherapy after surgery (55.7%), and had chronic diseases (23.1%), which may have increased their stress levels due to their inability to fulfill their roles and responsibilities expected by society.

### Gender role stress according to women's sociodemographic and breast cancer-related characteristics

In our study, it was determined that women with breast cancer who had bachelor's degrees, were employed, had higher income than expenses, had a nuclear family type, lived in a province, and whose spouses had bachelor's degrees experienced high levels of gender role stress. In a study, it was reported that women's education affected gender role stress [9]. In a study conducted with women with gynecologic cancer, most of whom were over the age of 50, married, and had a nuclear family type, it was found that women exhibited both positive and negative emotions, had limitations in daily life activities, their roles in couple/family were affected, and they experienced problems related to acceptance, emotional avoidance and spirituality [29]. While patriarchal values continue to have a deep and lasting influence in Turkish society, an increase in the influence of modern values is also observed [30]. This change in the social structure may have increased the stress related to gender roles in women with high education and income, working, living in the city, and having a nuclear family type due to the continued effects of patriarchal values. In addition, the negative effects of the disease process, such as the inability to fulfill daily life activities and withdrawal from working life, may have also affected women's gender role stress.

### Psychological distress levels in women with breast cancer

Psychological distress is a state of emotional suffering usually characterized by symptoms of depression and anxiety. Cancer is a chronic medical condition that is often perceived as a serious life-threatening illness and has significant effects on patients' physiological and psychological states. More than 25% of women with breast cancer report psychological symptoms [31]. The breast is one of the defining features of the female body, and the loss of the breast after breast cancer surgery is considered a traumatic psychological experience [24]. In this study, it was determined that women with breast cancer experienced high levels of psychological distress. In addition, it is a remarkable finding that this level is included in the classification of possible severe mental disorders. Studies have similarly reported that women with breast cancer experience high levels of psychological distress [24, 32, 33]. The increase in both the incidence and survival rate of breast cancer in Türkiye in recent years [30] is thought to have caused more and more women to live with the psychological repercussions of the disease and its treatment. In this context, the psychological needs of women after mastectomy have been revealed, and it is important to provide psychological support services.

### Levels of psychological distress according to sociodemographic and breast cancer-related characteristics of women

In the literature, some factors such as living in rural areas, chronic disease, religion, race, or having anxious characteristics at the time of diagnosis have been reported to be associated with psychological distress in women with breast cancer [31, 34]. In this study, it was determined that women with breast cancer who had bachelor's degrees, were retired, had a nuclear family type, lived in a province, had spouses with bachelor's degrees, had no chronic disease, and did not experience domestic intervention experienced high levels of psychological distress. In a study conducted with women with breast cancer, Berhili et al. (2019) found that inadequate social and family support and life difficulties (emotional, financial, or occupational) were associated with high psychological distress. However, education, place of residence, and occupation were not associated with psychological distress [24]. In a study, it was determined that women with breast cancer who had an underlying disease were more likely to experience psychological distress [34]. Our findings are similar to these studies.

In our study, it was determined that women with breast cancer who were in the first stage of cancer, who had a partial mastectomy, who did not receive any treatment other than surgery, and whose frequency of physician visits was less than one-year experienced high levels of psychological distress. Kaminska et al. found statistically significant differences between those who had undergone mastectomy and those who had undergone conservative surgery in their evaluations of "self-image" [25]. In a study, it was found that women with breast cancer who were newly diagnosed and who had just started the treatment process had higher levels of depression and stress [35]. Although our findings are consistent with previous studies, women's inability to adapt to the stress factors in life (chronic disease, income level), financial expenditures for treatment, and inability to cope with problems effectively may have increased their psychological distress levels.

### The relationship between gender role stress experienced by women with breast cancer and psychological distress

In our study, gender role stress experienced by women with breast cancer was not significantly associated with psychological distress. In contrast to our findings, a study found that young women with breast cancer who were more supportive of gender socialization had low psychological well-being, while women who resisted traditional gender role expectations, objectification pressures, and other social discourses had high psychological well-being [3]. In another study, it was reported that young women had low psychosocial well-being after breast cancer experience with their changing bodies, disease, and gender-related embodied identities [36]. In Frost's study, it was found that biographical distortion increased and body image decreased in women with breast cancer who defined themselves with moderate to high levels of feminine gender role orientation [19]. In a study by Li et al. (2017), it was determined that depression and physical symptoms associated with breast cancer treatment increased in young women who internalized more cultural stereotypes of femininity [37]. A recent meta-analysis revealed that contrary to studies in the literature, some women with breast cancer reported a positive sense of growth and development and new life after treatment [38]. In another study conducted with women with gynecologic cancer, it was found that psychological distress was significantly and positively associated with gender role attitude [27]. Emotional functioning can be influenced by gender dimensions in several ways. Research indicates that societal expectations and gender norms can shape how individuals cope with cancer and seek emotional support [39]. Women generally report greater use of emotional and social support networks [15]. A recent systematic review on coping strategies among patients with colorectal cancer reported that women seem to prefer using more adaptive coping strategies, switching between emotion-focused coping and problem-focused coping, to manage the various stressors that living with colorectal cancer entails [39]. One study reports that cultural family values, such as being protective and resourceful, can serve as powerful social motivators for coping with caregiving challenges and act as a protective buffer (e.g., suppressing cancer-related worries and denying partners' concerns) [6]. The endorsement of "feminine care" norms (e.g., "women are natural caregivers") can position women as "emotional nurturers of others" and, as a result, lead them to use protective buffering to minimize the burden on their families [40]. As stated in the literature, the fact that this study was conducted with women with breast cancer, who belong to a gender that can see the social values in our culture as a protective buffer to cope with cancer-related stress and difficulties, can share their thoughts and feelings with others more easily, and can receive more support from friends, may have influenced the lack of a relationship between gender role stress and psychological distress. Moreover, in this study, the fact that gender role stress was not associated with psychological distress may have been influenced by the fact that the mean age of the women was approximately 50 years, which differs from the studies in the literature; more than half of the women were housewives and therefore did not engage in employment that imposed responsibilities tied to any place or anyone else; 71.6% of the women did not lose both breasts, making the loss less visible on the body; and 71.6% of the women had more social activities they could engage in because they lived in urban areas. In addition, since Turkish society has a patriarchal culture, women's internalization of gender roles may not have affected their psychological well-being.

### Research perspectives and recommendations

The breast is closely linked to female identity and feminine gender role. Women's gender role plays an important role in the woman's body image and self-perception, and therefore in her psychological well-being, after treatment. Our data reveal the importance of assessing psychological health as well as gender role stress in post-mastectomy women with breast cancer. In this context, the findings of this study encourage researchers to focus on the effects of gender roles and the stress associated with these roles on psychological distress in women with breast cancer. In addition, since our country is a multicultural country, health professionals should conduct research on the traditional, stereotypical roles attributed to women and how these roles affect women's health.

Gender-related roles, norms, and stresses change by generation, and they are often deeply ingrained in social and cultural practices. These roles and norms are not inherently negative but offer a framework for understanding differences and similarities in social expectations and societal positions between people based on their gender identities. Moreover, gender-based roles and stresses vary according to socioeconomic factors. Socioeconomic factors such as age/generation, gender, chronic diseases, family structure, education level, and place of residence cannot be considered independently of gender role stress and psychological well-being [10]. Future studies should be conducted with more heterogeneous samples and larger sample sizes to increase the power of the studies and the accuracy of the results. This study encourages healthcare professionals to be aware that gender-based social expectations, norms, and stress-related behaviors can both facilitate and hinder their ability to effectively cope with cancer, and that these behaviors can affect patients' psychological well-being and, consequently, their quality of life. In this context, it is crucial for healthcare professionals (doctors, nurses, etc.) to reevaluate or reformulate restrictive concepts such as femininity in order to address the specific needs of women with breast cancer and provide personalized support.

### Study limitations

The limitations of the study are that it is a single-center, cross-sectional descriptive study and that it includes only women with breast cancer who had mastectomy, who attended the hospital where the study was conducted, and who agreed to participate in the study. Therefore, the results of the research cannot be generalized to the entire population. Furthermore, the inability to control for numerous variables such as women's cultural and religious beliefs and communication differences is another limitation of the study.

## Conclusion

This study found that women with breast cancer experience high levels of stress related to their social role. It was also determined that women experience high levels of psychological distress. In our study, it was observed that socioeconomic factors such as education, income level, family type, place of residence, and presence of chronic disease affect gender role stress and psychological distress.

Our data reveal the importance of assessing psychological health as well as gender role stress in post-mastectomy women with breast cancer. Health professionals (nurses, doctors, etc.) should consider psychological challenges and coping strategies as well as gender roles when evaluating women with post-mastectomy breast cancer holistically in terms of health.

## Data Availability

The data that support the findings of this study are available on request from the corresponding author.
